# Aetiology of non-malaria acute febrile illness fever in children in rural Guinea-Bissau: a prospective cross-sectional investigation

**DOI:** 10.3389/fepid.2024.1309149

**Published:** 2024-03-21

**Authors:** Rui Gutierrez, Mariana Landa, Masse Sambou, Hubert Bassane, Ndongo Dia, Alfa Saliu Djalo, Chiara Domenichini, Gamou Fall, Martin Faye, Ousmane Faye, Maria-Dolores Fernandez-Garcia, Laurence Flevaud, Jerlie Loko, Oleg Mediannikov, Valerie Mize, Kader Ndiaye, Mbayame Niang, Didier Raoult, Merce Rocaspana, Susana Villen, Amadou Alpha Sall, Florence Fenollar

**Affiliations:** ^1^ Médecins Sans Frontières, Barcelona Athens Operational Centre, Barcelona, Spain; ^2^ Vitrome, Aix Marseille Univ, Dakar, Senegal; ^3^ Institute de Recherche Pour le Development, IHU Méditerranée Infection, Dakar, Senegal; ^4^ Virology Pole, Institut Pasteur Dakar, Dakar, Senegal; ^5^ Vitrome, Aix Marseille Univ, Marseille, France; ^6^ Institute de Recherche Pour le Development, IHU Méditerranée Infection, Marseille, France

**Keywords:** fever, paediatrics, non-malaria acute febrile illness (NMAFI), West Africa, aetiology

## Abstract

**Background:**

With growing use of parasitological tests to detect malaria and decreasing incidence of the disease in Africa; it becomes necessary to increase the understanding of causes of non-malaria acute febrile illness (NMAFI) towards providing appropriate case management. This research investigates causes of NMAFI in pediatric out-patients in rural Guinea-Bissau.

**Methods:**

Children 0–5 years presenting acute fever (≥38°) or history of fever, negative malaria rapid diagnostic test (mRDT) and no signs of specific disease were recruited at the out-patient clinic of 3 health facilities in Bafatá province during 54 consecutive weeks (dry and rainy season). Medical history was recorded and blood, nasopharyngeal, stool and urine samples were collected and tested for the presence of 38 different potential aetiological causes of fever.

**Results:**

Samples from 741 children were analysed, the protocol was successful in determining a probable aetiological cause of acute fever in 544 (73.61%) cases. Respiratory viruses were the most frequently identified pathogens, present in the nasopharynx samples of 435 (58.86%) cases, followed by bacteria detected in 167 (22.60%) samples. Despite presenting negative mRDTs, *P. falciparum* was identified in samples of 24 (3.25%) patients.

**Conclusions:**

This research provides a description of the aetiological causes of NMAFI in West African context. Evidence of viral infections were more commonly found than bacteria or parasites.

## 1 Introduction

Malaria is the most prevalent cause of acute undifferentiated fever in children in West Africa (1). As the incidence of the disease decreases in the continent (1–4), and the use of parasitological tests increases (5–7), many patients who previously would be treated for malaria are now identified as having other diseases. Appropriate management of these patients continues to challenge healthcare providers (8, 9).

Malaria rapid diagnostic tests (mRDTs) are a vital part of the strategy to extend access to diagnosis in areas where good-quality microscopy cannot be maintained (10); their performance is directly related to the parasite density, with a recent study indicating sensitivity above 95% if density ≥400 parasites/µl and approaching 100% when the density reaches 4,000 parasites/µl, negative predictive value of 88% in the rainy season and 95% in the dry season (11).

Despite the good performance and availability of mRDTs, there is evidence of antimalarials prescribed for patients with negative mRDTs, undermining the benefits of diagnostic screening (12–14). In some contexts, the decrease in antimalarial consumption after introduction of mRDTs was accompanied by increased antibiotic use (15–17), raising concerns towards potential resistance.

Recent studies have investigated other potential causes of fever in hospitalized patients (18, 19) and in the community (20, 21), but few investigated this issue in West Africa (22–25), which has a unique hyperendemic, seasonal pattern of malaria incidence (2).

## 2 Materials and methods

### 2.1 Study design

Prospective cross-sectional study, designed to identify aetiology of non-malaria acute febrile illness (NMAFI) in children younger than five. Patients whose caretakers agreed to participate had clinical history recorded and bodily samples collected and tested in reference laboratories to detect acute infection of 38 different pathogens that could cause fever.

### 2.2 Setting

The research occurred in Bafatá (Guinea-Bissau), located 300 km from the capital. Guinea-Bissau is amongst the world's poorest and most fragile countries (26); it has a hyper-endemic malaria transmission pattern, similar to other countries of the sub-Sahel region, where malaria transmission is intense but seasonal (27). Médecins Sans Frontières (MSF) was present in Bafatá from 2014 to 2018, developing projects to reduce child morbidity and mortality. At the time, there were 5 functioning health facilities in the district but due to 2 of them being relatively small, enrolment took place at the outpatient clinic in only 3 of the facilities: Bafatá Regional Hospital, Tan-Tan Cossé and Cotumboel health centres.

### 2.3 Participants

Inclusion criteria: children under five years that attended the outpatient clinic in any of 3 health facilities presenting acute fever (axillary temperature ≥38°C) or history of fever for less than 14 days, with no signs or symptoms which would exclude Malaria as a diagnostical hypothesis and negative malaria mRDT (HRP2/pLDH, SD-Bioline®) were invited to participate in the research.

Exclusion criteria: chronic fever (lasting more than 14 days) or any sign or symptom that would exclude malaria as a differential diagnosis hypothesis. The research team actively investigated for franc rhinorrhoea, cough, expectoration, haemoptysis, wheezing, any abnormal respiratory sound, stridor, pharyngitis, otorrhoea, sore throat, melena, rectal bleeding, abdominal cramps, any symptom of the reproductive, neurologic or urinary systems, infected wounds, arthritis and oedema of the articulations.

Signs and symptoms considered compatible with malaria clinical presentation (28–30) (shivering, chills, vomiting, diarrhoea, convulsions, pallor, palpable spleen, reduced feeding or increased respiratory rate) would not exclude a child from participating.

Investigators explained the nature of the study, procedures and possible outcomes to parents/caretakers of children that met the inclusion criteria and written informed consent forms in accessible language were signed by both parties; a copy was provided. Two independent ethics committees approved the protocol and procedures, MSF's (ID1530) and Guinea-Bissau's INASA (0013/2015), which follow principles of the Declaration of Helsinki.

### 2.4 Controls

Due to logistic challenges, the DNA extraction from blood samples was performed in 2 stages, the first of which had to be done at the Bafatá regional hospital (see chapter 2.5 Variables); as this was the first time such procedures took place in the laboratory of that regional hospital, it was opted to recruit 69 children as controls for assessing the quality of the local sample management procedures. Children between 0 and 5 years that attended the facilities for reasons other than being sick, either for vaccination or accompanying patients (siblings) were invited to participate in the research as controls. They followed the same procedures as enrolled subjects, being tested twice with mRDT, assessed for clinical signs and symptoms, and interviewed. However, as naropharingeal and stool samples were not manipulated at the local laboratory, samples collected from controls were limited to blood for bacterial analysis.

### 2.5 Variables

Identification, socio-economic variables, medical history and details of the current clinical episode were recorded through a structured interview with caretakers; 2 blood samples and pooled nasal and throat swabs were collected from the children for microbiologic tests. Stool samples were collected only from subjects presenting diarrhoea.

The choice of pathogens was based on previous experience of MSF and national medical staff working in Guinea-Bissau and other countries in the region and on literature review (22–24, 31–33).

One of the blood samples was collected for identification of bacteria (*Anaplasma* spp, *Bartonella* spp, *Borrelia* spp, *Coxiella burnetii*, *Leptospira* spp, *Rickettsia* spp, *Salmonella enterica Typhi* and *Paratyphi*, *S. aureus*, *S. pneumonia*, *S. pyogenes* and *T. whipplei*), parasites (*Mansonella* spp) and protozoa (*Plasmodium falciparum*, *vivax*, *ovale* and *malariae*) through PCR. For this sample, DNA extraction was performed in two stages, using an EZNA tissue DNA kit (Omega Bio-tek Inc., USA); the first stage was performed in the laboratory of the Bafatá Hospital, following the manufacturer's instructions until the pre-elusion phase. Columns with the bound and dried DNA were stored between 4°C and 8°C and transferred to the Vitrome/IRD laboratory in Dakar under a cold chain, where the last steps of DNA extraction (elusion) were performed. The quality of the DNA extraction was controlled by a quantitative real-time PCR targeting human β-actin; samples positive for β-actin were included in the data analysis (32, 33).

The remainder of the samples (a second blood sample, nasopharyngeal swab and stool) were shipped to Institut Pasteur Dakar under a cold chain (between +4°C and +8°C), where RNA was extracted from centrifuged blood samples using the QIAamp® Viral RNA mini kit (QIAGEN, Germany). Sera were analyzed by Elisa and RNA extracts and tested by differential RT-qPCR assays using the QuantiTect Virus Kit (QIAGEN, Germany) for detection of IgM antibodies and genome of 7 arboviruses: Dengue, West Nile, Zika, Chikungunya, Yellow Fever, Rift Valley Fever and Crimean-Congo haemorrhagic fever viruses.

For viral respiratory pathogens detection from the nasopharyngeal samples, the Anyple® II RV16 Detection kit (South Korea) was used, which enabled simultaneous detection of several respiratory viruses: influenza A virus, influenza B virus, respiratory syncytial virus A and B, adenovirus, metapneumovirus, coronavirus 229E, NL63 and OC43, parainfluenza virus 1–4, rhinovirus A/B/C, enterovirus and bocavirus. Once in the laboratory, specimens were processed immediately for virus detection, identification, and characterization.

Stool specimens were diluted in phosphate-buffered saline and centrifuged twice. Nucleic acids were extracted from both stool supernatants and nasopharyngeal swabs using the QIAamp® Viral RNA kit (QIAGEN, USA) and analyzed by one-step multiplex RT-PCR assay using the Allplex® GI-Virus kit (South Korea) for detection of enteric viruses. The methodology allows the simultaneous detection of 6 enteric viruses belonging to genogroup (GI) and (GII) of noroviruses and the rotavirus, adenovirus, astrovirus and sapovirus genus.

As PCR is designed to amplify genetic material from tiny amounts, it is considered extremely sensitive, so sample contamination with minimal amounts of extraneous genetic material can produce false-positive results. It is also considered highly specific; a recent study indicated the sensitivity and specificity of PCR both to be 90% when compared to culture, with the relevant remark that it could detect infectious organisms not identified by blood culture (34).

Information regarding the initial diagnostic hypothesis, initial treatment and final diagnosis was recorded from patients’ files. In cases where the laboratory diagnosis represented an important change in the course of treatment, patients were followed up with either a telephone call asking caretakers to bring the child to a health facility or a home visit by the MSF team.

### 2.6 Data sources

Socio-economic and clinical information were collected through structured interviews with the caretakers upon enrolment. Diagnostics were confirmed from the samples taken, laboratories which performed the analysis are part of the research team and shared the results directly.

### 2.7 Bias

To avoid selection bias, the research team worked closely with the clinical staff from the services in which patients were recruited to ensure that all patients fitting the inclusion criteria were invited to participate in the research.

### 2.8 Study size

As no epidemiological profile had previously been assessed in Guinea-Bissau, a hypothetical 50% incidence was applied to Yamene's sample size formula (with 95% confidence interval and 5% precision) to estimate the number of subjects to be recruited. The estimation was increased by 5% for potential refusals and samples not reaching laboratories in analysis conditions. Recruitment took place over 54 consecutive weeks, it was expected to recruit 404 children during the rainy season and 404 during the dry season, to allow for comparisons. However, by the end of the recruitment period only samples from 741 subjects had been analyzed; 245 recruited during the dry season and 496 during the rainy season.

### 2.9 Quantitative variables

Participants were categorized according to age, following MSF Paediatric Guidelines (0–5, 6–11, 12–23 and 24–59 months). Positive results were classified according to which sample was positive (blood, nasopharynx swap or stool) as well as the category of the pathogen (bacteria, virus, protozoa, filaria).

### 2.10 Statistical methods

Data were entered in Epi Data, version 3.8 (EpiData Association®) and analysed in STATA version 14 (StataCorp®). Most analyses consist of simple proportions of positive samples (i.e., samples which indicated the presence of a pathogen by total samples analysed); differences between groups were compared using Pearsons *χ*^2^ and *p*-values presented.

### 2.11 Patient and public involvement

The protocol addressed a common demand from healthcare providers, specifically community health workers (CHWs), to better understand diseases that affect the population. The CHWs are members of their communities and express the health care concerns of the population; the difficulty in elaborating differential diagnoses for children that present NMAFI is of concern. The final report of this research was translated into Portuguese and local languages and shared with local healthcare providers and community health workers.

## 3 Results

### 3.1 Descriptive data

Enrolment occurred over 54 consecutive weeks, from August 2016 to September 2017. There were 783 children enrolled, but 42 (5%) were excluded due to their samples arriving at the laboratory in improper conditions for analysis. The demographics and clinical characteristics of participants recorded on enrolment are presented in Table 1.

**Table 1:** Demographics and clinical characteristics of participants.

Demographics/clinical characteristics	*N*	%
Male	399	54%
Female	340	46%
Age category		
0–5 months	40	5%
6–11 months	133	18%
12–23 months	203	27%
24–59 months	363	49%
Health facility		
Bafatá hospital	392	53%
Tan-Tan cosse	170	23%
Cotumboel	177	24%
Season		
Dry	244	33%
Rainy	495	67%
Febrile on consultation	266	36%
Fever in the past 48 h	732	99%
Fever lasting >48 h	155	21%
Chills	37	5%
Sweat	140	19%
Headache	118	16%
Constant crying	111	15%
Vomiting	103	14%
Diarrhoea	118	16%
Decline to breastfeed	22	3%
Abnormal general aspect	44	6%
Asthenia	7	1%
Loss of appetite	81	11%
Weight loss	37	5%
Pallor	15	2%
Dehydrated	7	1%
Increased respiratory rate	0	0%

Socio-economic variables: 652 caretakers (88%) did not have formal employment, 332 (45%) of the main providers of the households are farmers and 89 (12%) have jobs related to local commerce.

Medical History: 85 children (11%) had received treatment recently, 69 received malaria intemitent preventive treatment during infancy (IPTi) and 16 received treatment with either simptomatics or oral rehidration salts—none had received antibiotics. Most children (62%) did not have complete vaccination status for their age.

### 3.2 Main results

Overall, samples from 741 children were analysed to detect the presence of 38 different pathogens which could be the potential cause of NMAFI. At least one pathogen was detected on samples of 544 (74%) children. Only 285 (38%) children had complete vaccination status for their age and the probability of having a positive diagnosis did not vary depending on the vaccination status (*p*-value = 0.441). Pathogens were categorized by the sample in which they were identified and grouped according to Table 2.

**Table 2:** Frequency of pathogens identified.

Sample/pathogens	Cases (*N*)	%
Blood samples		
Bacteria		
*Rickettsia felis*	33	5
*Coxiella burnetii*	91	12
*Tropheryma whipplei*	1	0
*Staphilococus aureus*	39	7
*Streptococus pneumonia*	3	0
*Salmonella enterica* (Paratyphi)	13	2
*Salmonella enterica* (Typhi)	15	2
Protozoa		
*Plasmodium falciparum*	24	3
Filarea		
*Mansonella pertans*	11	2
Virus	0	–
Nasopharyngeal Samples		
Virus		
*Influenza B*	38	5
*Influenza A-H3N2*	11	2
*Influenza A-H1N1*	25	3
*Rhinovirus*	159	23
*Coronavirus OC43/NL63/229E*	30	4
*Parainfluenza 1–4*	42	6
*Bocavirus*	27	4
*Respiratory syncytial virus*	32	4
*Enterovirus*	52	7
*Adenovirus*	157	21
Stool Samples		
Virus		
*Norovirus*	3	0
*Rotavirus*	6	1
*Astrovirus*	7	1

Percentages account for more than 100% due to patients with multiple positive samples.

Microorganisms investigated but not detected in any of the samples are: *Anaplasma, Bartonella, Leptospira interrogans, Borrelia crocidurae*, *S. pyogenes*, *P. vivax, P. ovale*, *P. malariae*, *Dengue, West Nile, Zika, Yellow Fever, Crimean Congo* and *Chikungunya* viruses (blood samples), *Metapneumorvirus* (nasopharyngeal samples) and *Adenovirus* and *Sapovirus* (stool samples).

**Figure 1:**
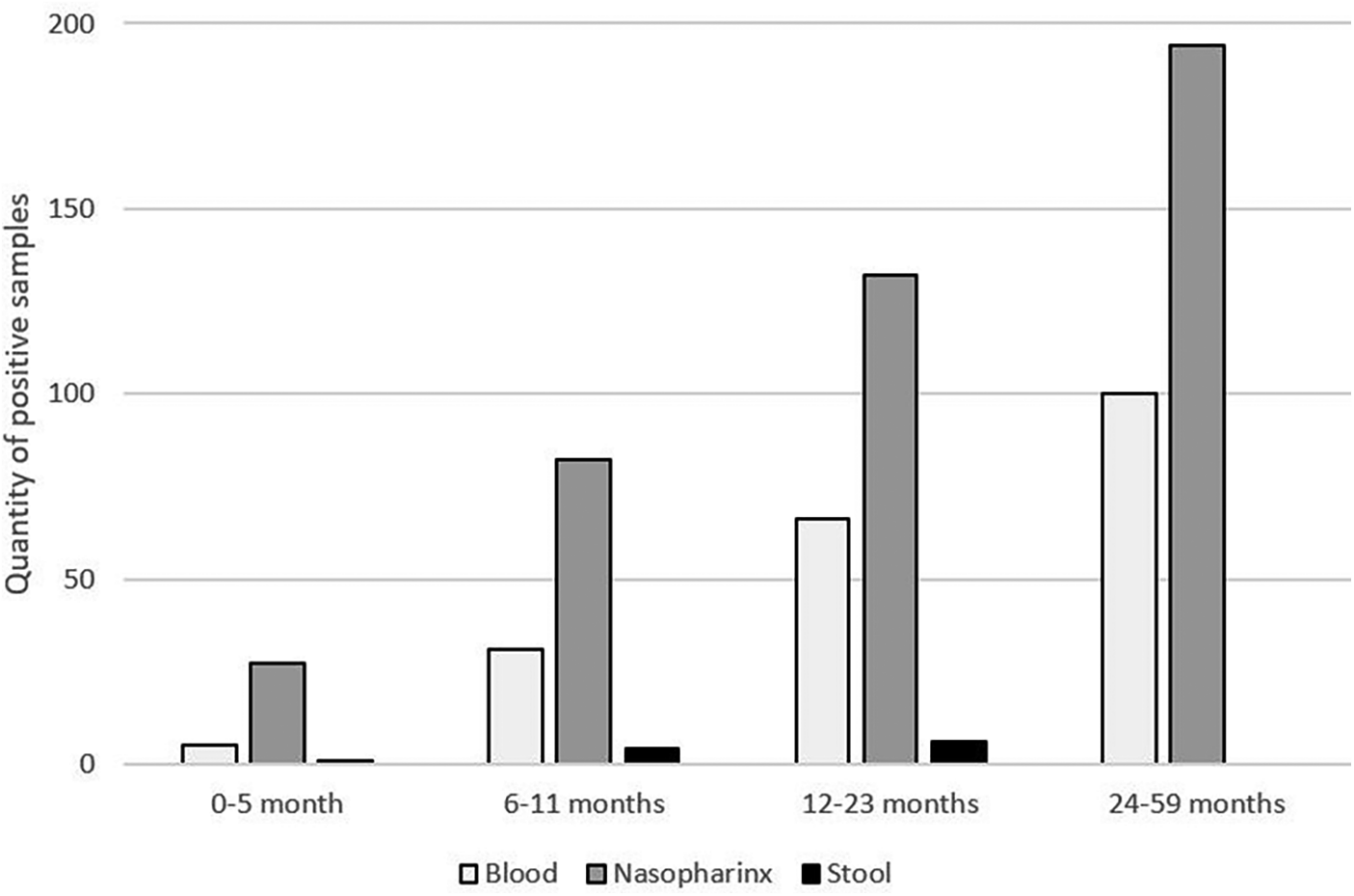
Quantity of positive samples detected per age category.

As illustrated in Figure 1, in all age categories, the microorganisms most frequently associated with NMAFI were viruses from nasopharyngeal samples, with at least one pathogen from this category being detected in samples of 59% of the patients, followed by microorganisms identified on blood samples (26% of patients) and stool samples, positive on samples of 1% of patients.

The most frequently identified viruses on nasopharyngeal samples were *Rhinovirus* and *Adenovirus*. Patients with at least one respiratory virus detected are, on average, slightly younger than patients whose pathogens were identified on other samples (median age 19 vs. 24 months, *p*-value = 0.0138). Regarding symptomatology, 42% of children with respiratory viruses were febrile upon consultation, significantly more than the children with other diagnoses (22%) (*p*-value <0.001). There was no statistically significant difference between the proportion of children diagnosed with respiratory viruses in the dry and rainy seasons (*p*-value = 0.730).

Bacteria were identified in blood samples of 167 individuals (23%), the most frequent *C. burnetii*, followed by *S. aureus* and *R. felis*—evidence of a Q fever epidemy, with 61 cases confirmed between October 2–16 and January 2017. All cases were confirmed by two different highly specific qPCRs and, in many cases, by amplification/sequencing of one or more variable spacers used for the following genotyping.

The research team tried to genotype the samples obtained during the study. Unfortunately, due to a low DNA concentration and a small sample quantity, it was only possible to identify the genotype in only one sample, genotype 6.

The cases of *C. burnetii* and *R. felis* occurred during the rainy season, with the peak incidence in November when the bacteria were identified as the cause of fever in 16% of the patients. Even though the cases of *C. burnetii* and *R. felis* seem to be clustered in time, this does not occur for geographical areas; the proportion of patients diagnosed with one of these bacteria was the same in all health centres (*p*-value = 0.200).

The second most frequent bacterial diagnostic found was *S. aureus*; these patients were more likely to have multiple diagnoses than those in which other pathogens were identified (64% and 36%, *p*-value = 0.001). There were no statistically significant differences in age (*p*-value = 0.785), and were more frequent during the dry season (*p*-value <0.001).

Negative Samples: 197 patients (27%) had negative results for all their samples. Patients in which no microorganism was identified are slightly younger than those in which at least one microorganism was identified; the mean age was 24.3 months for those in which no pathogen was identified compared to 22.0 months for those in which at least one pathogen was identified (*p*-value = 0.047). The analysis of the different signs and symptoms associated with NMAFI did not show any statistically significant differences between the two groups.

Multiple Positive Samples: the 544 individuals with positive samples, on average, had 1.51 (sd = 0.73) microorganisms identified, and 336 (62%) had only one positive sample. Up to 4 different pathogens were identified in the samples of a single individual; 109 co-infections (53%) occurred in the same diagnostic category group.

Despite having two negative mRDTs, *P. falciparum* was identified in 24 samples (3%); all of these patients had other pathogens identified in their samples, significantly more than patients with other diagnoses (*p*-value <0.001). Only 01 control (1.4%) had a positive finding, *P. falciparum*, with low parasitemia.

Upon receiving the laboratory diagnosis, investigators contacted the families by telephone. In cases in which the laboratory diagnosis posed a relevant change to the clinical conduct (specifically positive results for *C. burnetii*, *R. felix* or *S. Aureus*), caretakers were advised to take the child to the nearest health facility and home visits were conducted to the children whose families were not reachable by telephone.

In some cases, there were significant delays between enrolment and the laboratory diagnosis; 611 families were contacted (83%), eight children were no longer living in the region, four had presented with another clinical condition which was resolved before being contacted, one child had fever at the time the family was contacted, was taken to a health facility where she was diagnosed with malaria using mRDT (all samples from this child were negative at the moment of enrolment, leading the team to believe that she was infected between enrolment and the moment the family was contacted) and one patient deceased—a malnourished child, laboratory findings diagnosed with co-infection of *C. urnetiid* and *S. enterica* paratyphi.

## 4 Discussion

### 4.1 Key results

The protocol determined a probable cause of fever in 73% of children. Results draw a map of microorganisms causing non-malaria acute febrile illness in the Bafatá region of Guinea-Bissau. Except for the case of bacteria detected in the blood, there were no significant differences between pathogens identified in subjects enrolled during the dry and rainy seasons.

As anticipated by clinicians who work in this context, the most frequent cause of NMAFI is respiratory viruses that produce self-limited illness (35, 36). Considering that most patients (79%) attended the health facilities in less than 48 h from initial symptoms, it is not unusual that patients would not have developed other symptoms such as cough or rhinorrhea (which would exclude them from the protocol).

It was relieving that some diseases, such as Crimean-Congo haemorrhagic fever or Leptospirosis, which have typically high case fatality rates (37, 38), were absent in the samples of any children during the research. The pathogens capable of causing the most severe illness encountered were *C. burnetii*, *R. felis*, *S. aureus* and *P. falciparum*. Surprisingly, except for *P. falciparum*, no other disease transmitted through mosquito vectors (dengue, chikungunya, zika, yellow fever or other malaria) were detected during this research, indicating that, despite favourable climate conditions and the presence of the vector, these diseases were not circulating during the period of the research; this is different from the findings of recent investigations done in West Africa (20–22).

One of the striking issues in this study is the epidemy of Q fever and Rickettsia detected. This has been previously described in other research (32, 33, 39, 40). The clustering in time of the bacteria transmitted by vectors follows the narratives of the CHWs employed by MSF: they refer that the grass is not fit for cattle during the dry season, so they are only present during the rainy season. The CHWs also report that cattle often stay near the premises of the houses and that there is widespread consumption of raw milk in the markets, which could explain the incidence pattern (41). Even if incomplete, the genotyping data shows that most cases were genetically identical, so the epidemic may be due to a specific, not yet characterized genotype of *C. burnetii*. Interestingly, an extremely high endemicity of Q fever in Guinea-Bissau was first reported in 1952 by J. Tendeiro (42). It was the first major publication on Q fever in West Africa, with reports of infected multiple tick species and isolating several strains of *C. burnetii* from hard ticks, goat and cow milk.

Regarding *S. aureus*, considering patients were in good clinical condition upon consultation and follow-up, that they were more likely to present other co-infections and that minimal amounts of extraneous DNA would be sufficient to produce false-positive results, the investigators find it possible that these samples were contaminated, most likely in the first stage of DNA extraction, which took place in the laboratory of the Bafatá Hospital (this was the first time this procedure was performed in that laboratory). The distribution of cases enhances this hypothesis, with 10 (25%) of the samples in which S. aureus was detected having been collected/processed in only two days.

Despite two negative mRDT, *P. falciparum* was detected in samples of 24 patients (3%). Through qPCR, it was possible to determine that these patients had low parasitemia (below the detection capacity of mRDTs) and considering that the majority of them (88%) were diagnosed with co-infections, researchers find it reasonable to assume that the protozoa were not the cause of fever in these patients.

### 4.2 Limitations

The participation rate was not recorded. Potential participants were identified during the clinical consultations; only caretakers of children willing to participate attended the room where the research team worked.

The list of 38 pathogens investigated as potential causes of acute undifferentiated fever is extensive but not exhaustive and it is possible that microrganisms not included in it and other diseases represent some of the causes of NMAFI in the context. Also relevant to point out that patients that tested positive for malaria were not invited to participate in this research, thus not allowing for inferences regarding co-infections in malaria patients. Finally, as data collection took place a few years ago—before the Covid-19 pandemic—it is possible that the epidemiologic profile of NMAFI in children in this region has since changed.

### 4.3 Interpretation

Researchers hope this study will be useful for clinicians working in this region to determine the differential diagnosis in cases of children where malaria was suspected and excluded using mRDT. The knowledge generated by this research can contribute to increasing the quality of care for patients with NMAFI and provides scientific knowledge to combat the indiscriminate use of antimalarials and antibiotics.

### 4.4 Generalizability

It is likely that many contexts in the sub-Sahel region, particularly those that share malaria incidence patterns, will find similar pathogens distribution as causes of NMAFI in children under five years. However, further investigation of this nature is still needed in other regions with hyperendemic malaria transmission patterns to increase the external validity of the findings.

## Data Availability

The raw data supporting the conclusions of this article will be made available by the authors, without undue reservation.

## References

[1] Bhatt S Weiss DJ Cameron E Bisanzio D Mappin B Dalrymple U The effect of malaria control on plasmodium falciparum in Africa between 2000 and 2015. Nature. (2015) 526(7572):207–11. 10.1038/nature15535 26375008 PMC4820050

[2] Gething PW Patil AP Smith DL Guerra CA Elyazar IR Johnston GL A new world malaria map: plasmodium falciparum endemicity in 2010. Malar J. (2011) 10(1):1–6. 10.1186/1475-2875-10-378 22185615 PMC3274487

[3] Cotter C Sturrock HJ Hsiang MS Liu J Phillips AA Hwang J The changing epidemiology of malaria elimination: new strategies for new challenges. Lancet. (2013) 382(9895):900–11. 10.1016/S0140-6736(13)60310-4 23594387 PMC10583787

[4] Snow RW Guerra CA Noor AM Myint HY Hay SI. The global distribution of clinical episodes of plasmodium falciparum malaria. Nature. (2005) 434(7030):214–7. 10.1038/nature03342 15759000 PMC3128492

[5] Hanson K Goodman C. Testing times: trends in availability, price, and market share of malaria diagnostics in the public and private healthcare sector across eight sub-Saharan African countries from 2009 to 2015. Malar J. (2017) 16:1–6. 10.1186/s12936-017-1829-5 28526075 PMC5438573

[6] World Health Organization. Guidelines for the Treatment of Malaria. World Health Organization (2015).

[7] Murray CK Gasser RA Jr Magill AJ Miller RS. Update on rapid diagnostic testing for malaria. Clin Microbiol Rev. (2008) 21(1):97–110. 10.1128/CMR.00035-07 18202438 PMC2223842

[8] Reyburn H Mbatia R Drakeley C Carneiro I Mwakasungula E Mwerinde O Overdiagnosis of malaria in patients with severe febrile illness in Tanzania: a prospective study. Br Med J. (2004) 329(7476):1212. 10.1136/bmj.38251.658229.55 15542534 PMC529364

[9] Greenwood BM Bradley AK Greenwood AM Byass P Jammeh K Marsh K Mortality and morbidity from malaria among children in a rural area of the Gambia, West Africa. Trans R Soc Trop Med Hyg. (1987) 81(3):478–86. 10.1016/0035-9203(87)90170-2 3318021

[10] Cunningham J Jones S Gatton ML Barnwell JW Cheng Q Chiodini PL A review of the WHO malaria rapid diagnostic test product testing programme (2008–2018): performance, procurement and policy. Malar J. (2019) 18:1–5. 10.1186/s12936-019-3028-z 31791354 PMC6889598

[11] Bisoffi Z Sirima SB Menten J Pattaro C Angheben A Gobbi F Accuracy of a rapid diagnostic test on the diagnosis of malaria infection and of malaria-attributable fever during low and high transmission season in Burkina Faso. Malar J. (2010) 9(1):1–4. 10.1186/1475-2875-9-192 20609211 PMC2914059

[12] World Health Organization. WHO informal consultation on fever management in peripheral health care settings: a global review of evidence and practice.

[13] Joshi R Colford JM Jr Reingold AL Kalantri S. Nonmalarial acute undifferentiated fever in a rural hospital in central India: diagnostic uncertainty and overtreatment with antimalarial agents. Am J Trop Med Hyg. (2008) 78(3):393. 10.4269/ajtmh.2008.78.393 18337332

[14] Hamer DH Ndhlovu M Zurovac D Fox M Yeboah-Antwi K Chanda P Improved diagnostic testing and malaria treatment practices in Zambia. JAMA. (2007) 297(20):2227–31. 10.1001/jama.297.20.2227 17519412 PMC2674546

[15] D’Acremont V Kahama-Maro J Swai N Mtasiwa D Genton B Lengeler C. Reduction of anti-malarial consumption after rapid diagnostic tests implementation in Dar es Salaam: a before-after and cluster randomized controlled study. Malar J. (2011) 10(1):1–6. 10.1186/1475-2875-10-107 21529365 PMC3108934

[16] Mubi M Kakoko D Ngasala B Premji Z Peterson S Björkman A Malaria diagnosis and treatment practices following introduction of rapid diagnostic tests in Kibaha district, coast region, Tanzania. Malar J. (2013) 12:1–8. 10.1186/1475-2875-12-293 23977904 PMC3765530

[17] Mosha JF Conteh L Tediosi F Gesase S Bruce J Chandramohan D Cost implications of improving malaria diagnosis: findings from North-Eastern Tanzania. PLoS One. (2010) 5(1):e8707. 10.1371/journal.pone.0008707 20090933 PMC2806838

[18] Brent AJ Ahmed I Ndiritu M Lewa P Ngetsa C Lowe B Incidence of clinically significant bacteraemia in children who present to hospital in Kenya: community-based observational study. Lancet. (2006) 367(9509):482–8. 10.1016/S0140-6736(06)68180-4 16473125

[19] Punjabi NH Taylor WR Murphy GS Purwaningsih S Picarima H Sisson J Etiology of acute, non-malaria, febrile illnesses in Jayapura, Northeastern Papua, Indonesia. Am J Trop Med Hyg. (2012) 86(1):46. 10.4269/ajtmh.2012.10-0497 22232450 PMC3247108

[20] D'acremont V Kilowoko M Kyungu E Philipina S Sangu W Kahama-Maro J Beyond malaria—causes of fever in outpatient Tanzanian children. N Engl J Med. (2014) 370(9):809–17. 10.1056/NEJMoa1214482 24571753

[21] Crump JA Morrissey AB Nicholson WL Massung RF Stoddard RA Galloway RL Etiology of severe non-malaria febrile illness in Northern Tanzania: a prospective cohort study. PLoS Negl Trop Dis. (2013) 7(7):e2324. 10.1371/journal.pntd.0002324 23875053 PMC3715424

[22] Baba M Logue CH Oderinde B Abdulmaleek H Williams J Lewis J Evidence of arbovirus co-infection in suspected febrile malaria and typhoid patients in Nigeria. J Infect Dev Ctries. (2013) 7(1):51–9. 10.3855/jidc.2411 23324821

[23] Baba MM Saron MF Vorndam AV Adeniji JA Diop O Olaleye D. Dengue virus infections in patients suspected of malaria/typhoid in Nigeria. J Am Sci. (2009) 5(5):129–34.

[24] Tarnagda Z Cissé A Bicaba BW Diagbouga S Sagna T Ilboudo AK Dengue fever in Burkina faso, 2016. Emerg Infect Dis. (2018) 24(1):170. 10.3201/eid2401.170973 29260685 PMC5749475

[25] Dahmane A Van Griensven J Van Herp M Van den Bergh R Nzomukunda Y Prior J Constraints in the diagnosis and treatment of lassa fever and the effect on mortality in hospitalized children and women with obstetric conditions in a rural district hospital in Sierra Leone. Trans R Soc Trop Med Hyg. (2014) 108(3):126–32. 10.1093/trstmh/tru009 24535150 PMC4023273

[26] Barry BS. Conflict, Livelihoods, and Poverty in Guinea-Bissau. World Bank Publications (2007).

[27] World Health Organization. World Health Statistics 2010. World Health Organization (2010).

[28] Chandramohan D Carneiro I Kavishwar A Brugha R Desai V Greenwood B. A clinical algorithm for the diagnosis of malaria: results of an evaluation in an area of low endemicity. Trop Med Int Health. (2001) 6(7):505–10. 10.1046/j.1365-3156.2001.00739.x 11469942

[29] Bojang KA Obaro S Morison LA Greenwood BM. A prospective evaluation of a clinical algorithm for the diagnosis of malaria in Gambian children. Trop Med Int Health. (2000) 5(4):231–6. 10.1046/j.1365-3156.2000.00538.x 10810013

[30] Mwangi TW Mohammed M Dayo H Snow RW Marsh K. Clinical algorithms for malaria diagnosis lack utility among people of different age groups. Trop Med Int Health. (2005) 10(6):530–6. 10.1111/j.1365-3156.2005.01439.x 15941415 PMC3521057

[31] Fenollar F Mediannikov O Socolovschi C Bassene H Diatta G Richet H Tropheryma whipplei bacteremia during fever in rural West Africa. Clin Infect Dis. (2010) 51(5):515–21. 10.1086/655677 20658941

[32] Mediannikov O Fenollar F Socolovschi C Diatta G Bassene H Molez JF *Coxiella burnetii* in humans and ticks in rural Senegal. PLoS Negl Trop Dis. (2010) 4(4):e654. 10.1371/journal.pntd.0000654 20386603 PMC2850317

[33] Mediannikov O Socolovschi C Edouard S Fenollar F Mouffok N Bassene H Common epidemiology of Rickettsia felis infection and malaria, Africa. Emerg Infect Dis. (2013) 19(11):1775. 10.3201/eid1911.130361 24188709 PMC3837673

[34] Nguyen MH Clancy CJ Pasculle AW Pappas PG Alangaden G Pankey GA Performance of the T2Bacteria panel for diagnosing bloodstream infections: a diagnostic accuracy study. Ann Intern Med. (2019) 170(12):845–52. 10.7326/M18-2772 31083728

[35] Heikkinen T Järvinen A. The common cold. Lancet. (2003) 361(9351):51–9. 10.1016/S0140-6736(03)12162-9 12517470 PMC7112468

[36] Lorber B. The common cold. J Gen Intern Med. (1996) 11:229–36. 10.1007/BF02642480 8744881 PMC7089473

[37] Yilmaz GR Buzgan T Irmak H Safran A Uzun R Cevik MA The epidemiology of Crimean-Congo hemorrhagic fever in Turkey, 2002–2007. Int J Infect Dis. (2009) 13(3):380–6. 10.1016/j.ijid.2008.07.021 18986819

[38] Abela-Ridder B Bertherat E Durski K. Global burden of human leptospirosis and cross-sectoral interventions for its prevention and control. Conference PMA, Editor. Prince Mahidol Award Conference (2013).

[39] Vanderburg S Rubach MP Halliday JE Cleaveland S Reddy EA Crump JA. Epidemiology of *Coxiella burnetii* infection in Africa: a OneHealth systematic review. PLoS Negl Trop Dis. (2014) 8(4):e2787. 10.1371/journal.pntd.0002787 24722554 PMC3983093

[40] Prabhu M Nicholson WL Roche AJ Kersh GJ Fitzpatrick KA Oliver LD Q fever, spotted fever group, and typhus group rickettsioses among hospitalized febrile patients in northern Tanzania. Clin Infect Dis. (2011) 53(4):e8–15. 10.1093/cid/cir411 21810740 PMC3148261

[41] Kim SG Kim EH Lafferty CJ Dubovi E. *Coxiella burnetii* in bulk tank milk samples, United States. Emerg Infect Dis. (2005) 11(4):619. 10.3201/eid1104.041036 15829205 PMC3320343

[42] Tenedeiro J. Febre Q Bissau, Portuguese Guinea. Centro de estudos da Guiné Portuguesa. 340 p.

